# Thymic Acid

**Published:** 1869-01

**Authors:** 


*Thymic Acid.*—From our medical exchanges we learn that thymic acid, which is "obtained from the essential oil of thyme (*thymus vulgaris*) has been proposed as a substitute for carbolic acid or creosote. In its powerfully antiseptic and emits no disagreeable odor. In its concentrated form, it may take the place of nitrate of silver as an escharotic; as an antiseptic it should be dissolved in 100 parts of water with a little alcohol." As the odor of creosote and carbolic acid is very disagreeable to many patients, it would be well for dental practitioners to give this agent a trial.

